# A putative pyruvate transporter TaBASS2 positively regulates salinity tolerance in wheat via modulation of *ABI4* expression

**DOI:** 10.1186/s12870-016-0795-3

**Published:** 2016-05-10

**Authors:** Yang Zhao, Xinghui Ai, Mengcheng Wang, Langtao Xiao, Guangmin Xia

**Affiliations:** The Key Laboratory of Plant Cell Engineering and Germplasm Innovation, Ministry of Education, School of Life Science, Shandong University, 27 Shanda South Road, Jinan, Shandong 250100 China; Hunan Provincial Key Laboratory of Phytohormones and Growth Development, Hunan Provincial Key Laboratory for Crop Germplasm Innovation and Utilization, Hunan Agricultural University, Changsha, 410128 China

**Keywords:** ABI4, BASS2, Oxidative stress, ROS, Salinity tolerance, Wheat

## Abstract

**Background:**

High salinity adversely affects crop production. Pyruvic acid is the precursor of abscisic acid (ABA) and other chemicals that are synthesized in chloroplast, some of which are involved in the response to salt. The transportation of pyruvic acid into chloroplast is mediated by pyruvate transporters. However, whether pyruvate transporters are involved in salt response has not been studied so far. Here, we answered this issue by assessing the function of a wheat pyruvate transporter in salt response.

**Results:**

A pyruvate transporter *TaBASS2* was isolated from salt-tolerant wheat cultivar Shanrong 3. The expression of *TaBASS2* was induced by NaCl stress as well as H_2_O_2_ and ABA treatments. Constitutive expression of *TaBASS2* in *Arabidopsis bass2-1* mutant complemented the mevastatin-sensitive phenotype that reflects the deficiency of transporting pyruvic acid into chloroplast. Overexpression of *TaBASS2* enhanced salinity tolerance and reactive oxygen species scavenging in wheat. *Arabidopsis* constitutively expressing *TaBASS2* also exhibited enhanced tolerance to salinity and oxidative stress. In *Arabidopsis*, *TaBASS2* repressed the expression of *ABA INSENSITIVE 4* (*ABI4*), a node linking ABA signaling and plastid retrograde signaling pathways. However, the enhanced salinity tolerance of *TaBASS2* overexpression *Arabidopsis* was abolished when *ABI4* expression was restored to the level of wild-type through overexpressing *ABI4*.

**Conclusions:**

Our work demonstrates that *TaBASS2* enhances salinity tolerance of plants via modulating *ABI4* expression. This indicates that pyruvate transporters indeed participate in the interaction of plants with environmental stimuli.

**Electronic supplementary material:**

The online version of this article (doi:10.1186/s12870-016-0795-3) contains supplementary material, which is available to authorized users.

## Background

High levels of soil salinity impose osmotic stress and ion toxicity on plants, leading to cell damage and growth arrest. Most crop plants are sensitive to excess salt concentration in soil [1]. With the salt-affected farming land expanding nowadays (http://www.fao.org/soils-portal/soil-management/management-of-some-problemsoils/salt-affected-soils/more-information-on-salt-affected-sovils/en/), salinity stress has become one of the most serious limiting factors in crop production. As an urgent global challenge of food security occurs, it is of great importance to understand the mechanism underlying plant response to salinity stress and develop novel salinity tolerant crop cultivars.

In the past few decades, extensive studies on salinity stress in plants, especially in the model plant *Arabidopsis thaliana*, have uncovered a number of genes involved in plant salt tolerance [2, 3]. Both abscisic acid (ABA)-dependent and -independent signaling pathways play important roles during this process [4, 5]. ABA INSENSITIVE 4 (ABI4) is an AP2/EREBP transcription factor that functions as a positive regulator in the ABA signaling pathway during seed development and germination [6]. ABI4 participates in other aspects of plant development, including salinity response and retrograde signaling [7–11]. Under salinity stress, three loss-of-function mutations in *ABI4* conferred increased tolerance in both seedling and adult stage, while the transgenic plants overexpressing *ABI4* were hypersensitive to NaCl treatment [7, 11]. Further studies revealed that ABI4 negatively regulated the expression of a high affinity K^+^ transporter, *HKT1;1*, by directly binding to two ABI4-binding elements (ABE) in *HKT1;1*’s promoter region [11].

Besides osmotic stress and ion toxicity, high salinity condition also adversely affects photosynthesis, cellular energy depletion and redox homeostasis [12–14]. Production and accumulation of excess reactive oxygen species (ROS), such as superoxide (O_2_^-^) and hydrogen peroxide (H_2_O_2_), cause oxidative damages in apoplastic compartments and cellular membranes. ROS also function as signaling molecules to activate gene expression in nucleus [1, 15, 16], including a number of ROS scavenging genes, such as *CATALASE* (*CAT*) [17, 18]. In *A. thaliana*, a transient burst of ROS production follows the imposition of abiotic stresses, and any disruption to ROS synthesis has a negative effect on the plant’s growth and its ability in stress response [13].

Chloroplast is closely associated with salt response in plants. It is not only a factory for energy assimilation but also the site for synthesis of ABA and other important metabolites, for pyruvic acid serves as the precursor. The bile acid/sodium symporter 2 (BASS2) is responsible for pyruvate uptake into chloroplast [19]. In *Arabidopsis bass2-1* mutant, the plastidal isopentenyl diphosphate (IPP) synthesis is blocked, so the mutant seedlings exhibit increased sensitivity to mevastatin, an inhibitor of cytosolic IPP synthesis pathway [19].

As an important staple crop, bread wheat belongs to glycophytes and displays high sensitivity to excess soil salinity, while its halophytic relative tall wheatgrass (*Thinopyrum ponticum*) is able to grow at salt concentrations as high as in seawater. In our previous study, a salinity-tolerant introgression wheat cultivar Shanrong No. 3 (SR3) was bred using asymmetric somatic hybridization [20]. SR3 wheat plants exhibited high level of tolerance under osmotic and saline stresses, and better performance in removal of toxic substances [21, 22]. In a further transcriptomic study, we identified a putative pyruvate transporter gene, *TaBASS2*, was up-regulated by NaCl treatment in SR3 instead of salinity-sensitive JN177 [23]. Here, we isolated the *TaBASS2* sequence and characterized its role in salinity tolerance. Constitutively expressing *TaBASS2* enhanced the salinity tolerance in transgenic wheat and *Arabidopsis*. The ROS contents and scavenging activity were enhanced in the transgenic plants as well. Further experiments indicated *TaBASS2* positively regulates plant response to salinity stress by repressing *ABI4* expression.

## Results

### *TaBASS2* is induced by NaCl treatment

**Fig. 1:**
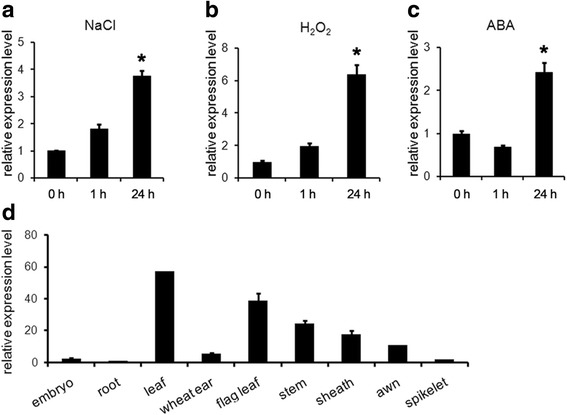
*TaBASS2* expression in wheat cv. SR3 plants treated with (**a**) 200 mM NaCl, (**b**) 100 μM ABA or (**c**) 10 mM H_2_O_2_, and in different tissues (**d**). Error bars represent the standard errors (*n =* 3), with each replicate comprising at least 12 plants. Columns labeled with an asterisk in (a-c) indicate significant differences from those at 0 h (*P <* 0.05, *t*-test). The expression levels were determined by RT-qPCR using *TaCyclophilin *as the internal control

To determine the expression pattern of *TaBASS2* under salinity stress, three-leaf-stage SR3 seedlings were treated with 200 mM NaCl solution and monitored *TaBASS2* expression in roots. As shown in Fig. 1a, *TaBASS2* was induced by two-fold as early as 1 h of the treatment, and the expression level increased up to four-fold at 24 h. The treatment with H_2_O_2_ or ABA also resulted in a similar induction of *TaBASS2* expression at 24 h (Fig. 1b, c). These results demonstrated that *TaBASS2* expression responded to high level of salinity as well as other stress signals. Various tissue types were also collected for another expression assay. The result showed that *TaBASS2* was transcribed in all the tested tissues, with higher expression levels in the green tissues (Fig. 1d).

To study the biological role of *TaBASS2* in plant response to salinity stress, the coding sequence (CDS) was cloned from a cDNA library constructed from the NaCl-treated SR3 seedlings. The resulting CDS was 1,242 bp in length, encoding a 413-aa putative BASS protein with eight transmembrane domains. The NCBI non-redundant protein sequence database was searched for the homologues, and 42 similar proteins across various organisms were found. The multi-alignment with five closest homologs showed that the most conserved regions resided in the eight transmembrane domains (indicated as TM1-TM8 in Additional file 1). A phylogenetic analysis clustered the wheat BASS amino acid sequence with those from monocots. The sequences from dicotyledonous species, including BASS2 (At2g26900; Additional file 1), clustered together, which together with the monocotyledonous cluster formed a BASS2 clade. Since the wheat BASS protein sequence shared a high similarity with BASS2, the gene was therefore named as *TaBASS2*, which was the first *BASS* gene cloned in bread wheat.

### *TaBASS2* complements *Arabidopsis bass2-1* mutant

**Fig. 2:**
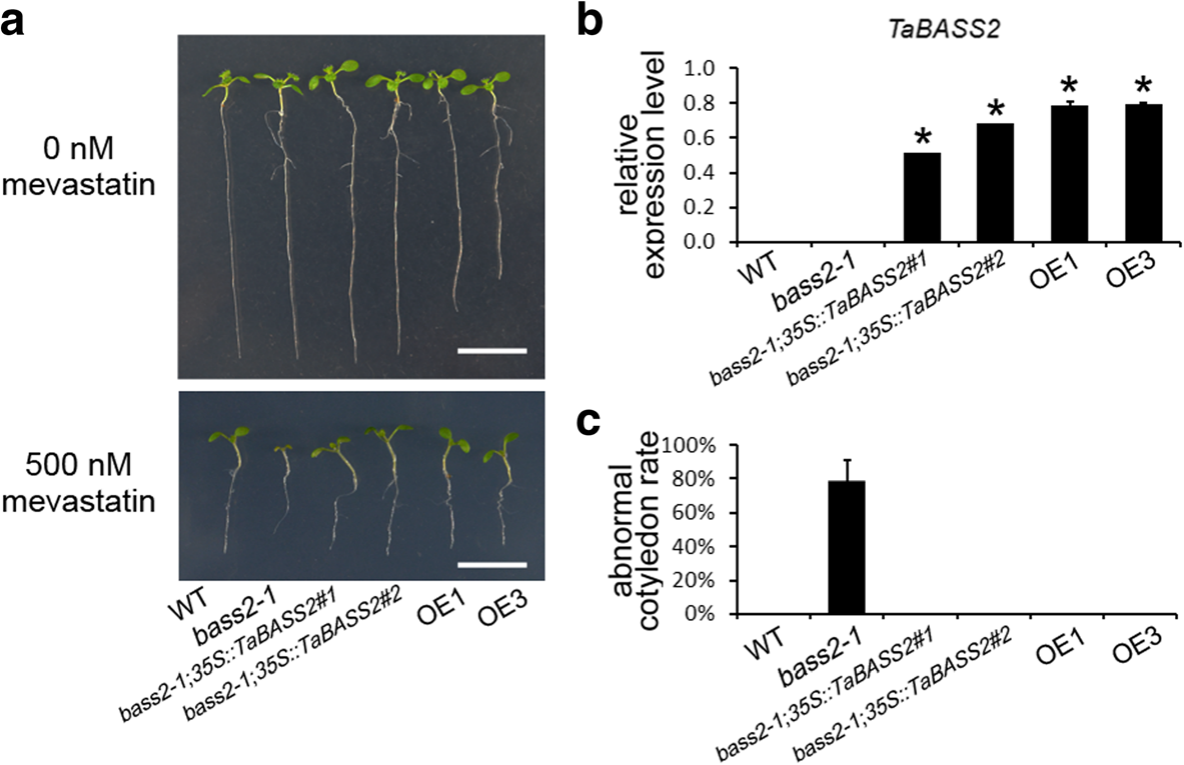
*TaBASS2* complements the *bass2-1* mutant*.* **a** The wild-type, *bass2-1*, *bass2-1;35S::TaBASS2* (#1 and #2)*,* and *35S::TaBASS2* (OE1 and OE3) plants after a seven-day exposure to 500 nM mevastatin. Bar = 1 cm. **b** *TaBASS2* expression in the wild-type, *bass2-1*, *bass2-1;35S::TaBASS2* and *35S::TaBASS2* lines. The expression levels were determined by RT-qPCR using *AtACT2* as the internal control. **c** The percentage of seedlings with abnormal cotyledons in the wild-type, *bass2-1*, *bass2-1;35S::TaBASS2* and *35S::TaBASS2* lines. Error bars in (b, c) represent the standard errors (*n =* 3), with each replicate comprising at least 12 plants; columns labeled with an asterisk indicate means differing significantly from *bass2-1* (*P <* 0.05, *t*-test)

*Arabidopsis* BASS2 is a sodium-dependent pyruvate transporter localized in plastid. Its knockout mutant in *Arabidopsis*, *bass2-1*, displays increased sensitivity to mevastatin [19]. To determine whether TaBASS2 has the same function as BASS2, the *Arabidopsis bass2-1* mutant was transformed with a *35S::TaBASS2* construct. Two transgenic lines with high *TaBASS2* expression levels, *bass2-1;35S::TaBASS2* #1 and #2, were selected to test the sensitivity to mevastatin (Fig. 2b). After a ten-day treatment with 500 nM mevastatin, the *bass2-1* seedlings showed severe abnormality in cotyledon, while the *bass2-1;35S::TaBASS2* seedlings developed a normal phenotype, the same as the wild-type *Arabidopsis* (Fig. 2a, c). This result demonstrated that *TaBASS2* completely complemented the mevastatin-sensitive phenotype of *bass2-1*. Moreover, the TaBASS2-GFP fusion protein was localized in plastid when transiently expressed in *Arabidopsis* mesophyll protoplasts (Additional file 2). These results together indicate that TaBASS2 is a genuine homolog of BASS2, which functions as a pyruvate transporter.

### Overexpressing *TaBASS2* enhanced salinity tolerance in wheat seedlings

**Fig. 3:**
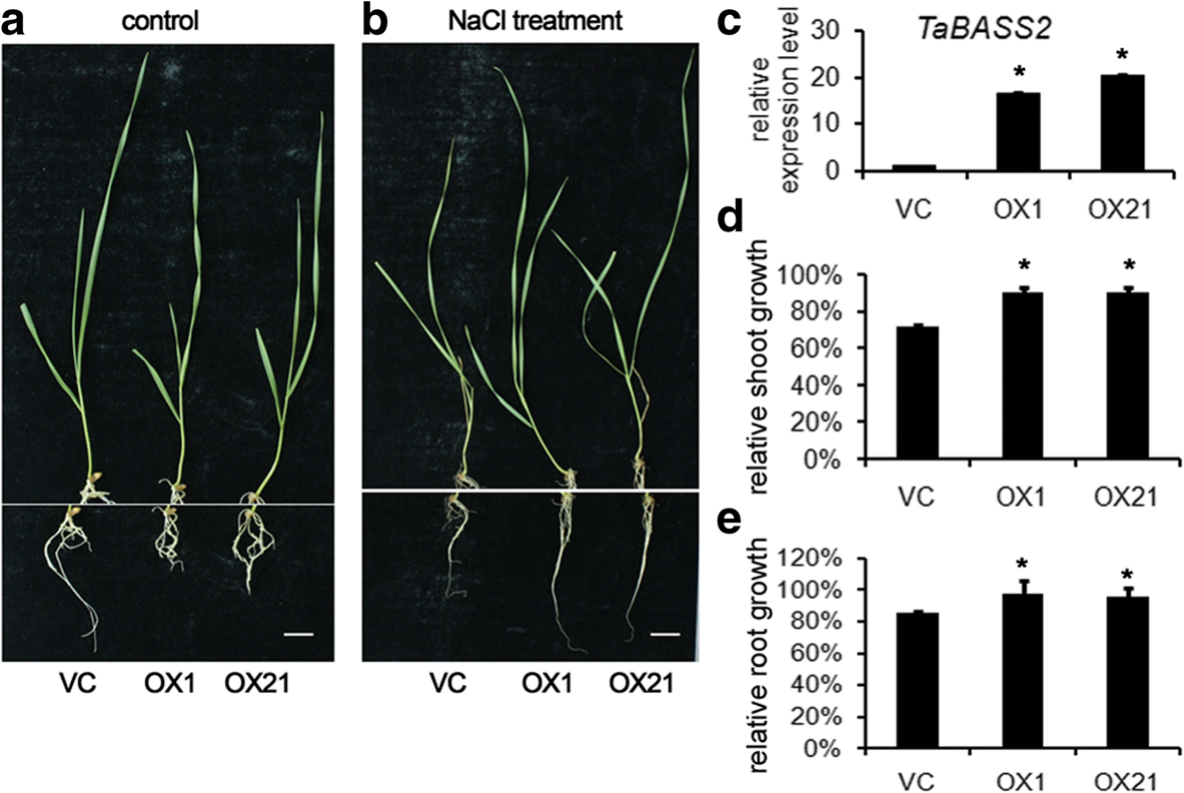
Constitutively expressing *TaBASS2* enhances salinity tolerance in wheat. **a**, **b** The vector control (VC) and two *Ubi::TaBASS2* wheat seedlings (OX1 and OX21) after an eight-day mock or NaCl treatment. Bar = 1 cm. **c** *TaBASS2* expression in the genotypes listed in (**a**). The expression levels were determined by RT-qPCR using *TaCyclophilin* in wheat as the internal control. Error bars represent the standard errors (*n =* 3), with each replicate comprising at least 12 plants. **d**, **e** Relative shoot and root growth in the genotypes listed in (**a**). Error bars represent the standard errors (*n* = 3), with each replicate comprising at least 30 plants. Columns labeled with an asterisk indicate means differing significantly from the VC result (*P <* 0.05, *t*-test)

The induction of *TaBASS2* by the NaCl treatment suggests its role in plant response to salinity stress (Fig. 1a). To characterize its function in wheat, *TaBASS2* was overexpressed in a salinity sensitive wheat cultivar YM20. From 34 independent transgenic lines, two lines (OX1 and OX21) with high transgene expression were selected for further experiments (Fig. 3c). Both OX1 and OX21 transgenic lines developed shorter shoots and roots than the empty vector control (VC) seedlings (Additional file 3). Thus, to determine the extent of salinity tolerance, the shoot/root lengths of NaCl-treated plants were normalized by dividing the shoot/root lengths of untreated plants to get relative shoot/root growth rates. After an eight-day NaCl treatment, the relative shoot growth in the VC line was around 72 % of that under the control condition, and the relative root growth was around 85 % compared with that under the control condition, displaying suppression in plant growth under high NaCl concentration. However, in the NaCl-treated OX1 and OX21 plants, both the shoot and root growth showed less suppression compared with the VC plants. After the NaCl treatment, the relative shoot growths were 98 % and 95 % compared with the control in OX1 and OX21, respectively, and the relative root growths were 90 % and 91 % of those under the control condition (Fig. 3d, e). These phenotypic results demonstrated that constitutively expressing *TaBASS2* relieved the growth suppression imposed by high saline concentration, thus enhanced salinity tolerance in the transgenic wheat plants.

### The constitutive expression of *TaBASS2* in *A. thaliana* enhanced the salinity tolerance

**Fig. 4:**
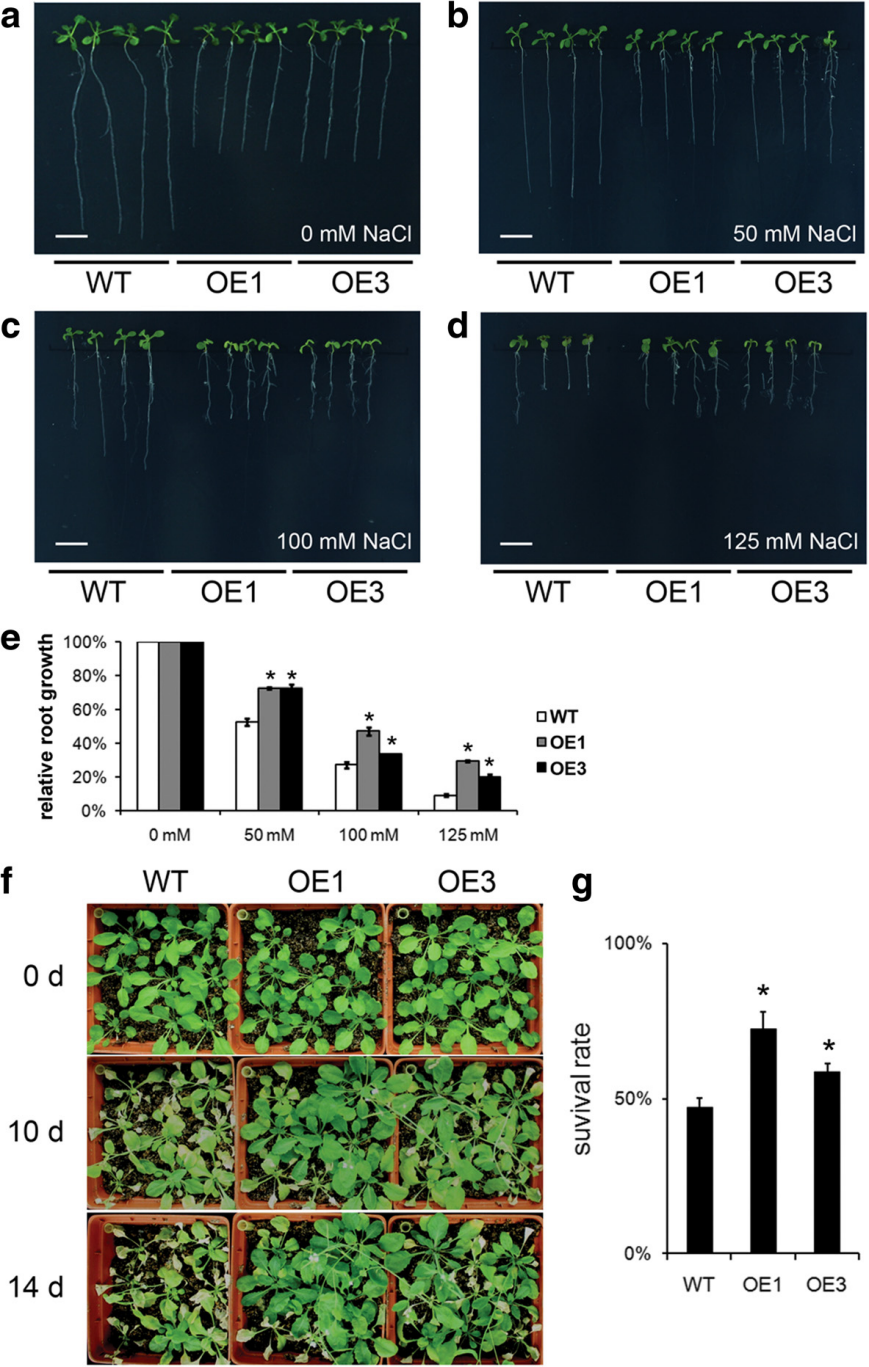
Constitutively expressing *TaBASS2* enhances the salinity tolerance in *Arabidopsis.* **a**-**d** The wild-type seedlings and two *35S::TaBASS2* transgenic lines (OE1 and OE3) after a ten-day treatment with 0, 50, 100 or 125 mM NaCl. Bar = 1 cm. **e** Relative root growth of the wild-type and OE plants treated with 0, 50, 100 or 125 mM NaCl. **f** The four-week-old soil-grown wild-type and OE plants 0, 10 and 14 days after NaCl treatment. **g** Plant survival rates measured at 14 day after NaCl treatment. Error bars in (**e**, **g**) represent the standard errors (*n* = 3), with each replicate comprising at least 30 plants. Columns labeled with an asterisk indicate means differing significantly from the WT result (*P <* 0.05, *t*-test)

To further reveal its biological role in salinity tolerance, transgenic *Arabidopsis* plants constitutively expressing *TaBASS2* driven by a 35S promoter were generated, from which two independent lines with high *TaBASS2* expression levels, OE1 and OE3, were selected for further experiments (Fig. 2b). The wild-type and transgenic lines were treated with a series concentrations of NaCl to determine their salinity response. Given that the roots of transgenic *Arabidopsis* seedlings were shorter than those in the wild-type (Fig. 4a, Additional file 4), the relative root growth rate was calculated to determine their salinity tolerance. Under a 50 mM NaCl treatment, the relative root growth rates of wild-type plants were 53 % of those under the control condition, while the relative root growth rates in the both transgenic plants were 73 % of those under the control condition (Fig. 4b, e). When the NaCl concentration was raised to 100 mM, the relative root growth in the wild-type was 27 %, but 47 % and 33 % in OE1 and OE3, respectively (Fig. 4c, e); under the 125 mM NaCl treatment, the relative root growth was about 9, 29 and 20 % in the wild-type and the two OE lines, respectively (Fig. 4d, e). This result demonstrated the *Arabidopsis* seedlings with constitutive expression of *TaBASS2* exhibited enhanced tolerance to salinity stress.

A further experiment was carried out to determine the salinity tolerance in the adult transgenic plants constitutively expressing *TaBASS2*. Four-week-old soil-grown wild-type and two *35S::TaBASS2* lines were treated with increasing concentration of NaCl for 14 days, and the survival rates were scored at the 14th day following the treatment (See Methods). As shown in Fig. 4f, after a ten-day NaCl treatment, the leaves of wild-type plants developed chlorosis, while the leaves of OE plants showed less severe response. At the 14th day after treatment, more than half of the wild-type plants died of high levels of soil salinity, while the majority of OE plants kept alive and even grew bigger (Fig. 4f). Consistent with the phenotypic response, the survival rate was 47 % in the wild-type plants at the 14th day after treatment, while it was 72 % and 58 % in the OE1 and OE3 lines respectively, significantly higher than that in the wild-type (Fig. 4f, g). It is also interesting to mention that *bass2-1* mutant showed no difference in the survival rate under NaCl treatment, probably due to the cytosolic IPP pathway (Additional file 5). These results showed that the *35S::TaBASS2* transgenic plants exhibited enhanced tolerance to NaCl treatment in both seedling and adult stages, demonstrating that constitutive expression of *TaBASS2* enhanced salinity tolerance in *Arabidopsis*.

### The transgenic *Arabidopsis* constitutively expressing *TaBASS2* showed the enhanced oxidative tolerance

**Fig. 5:**
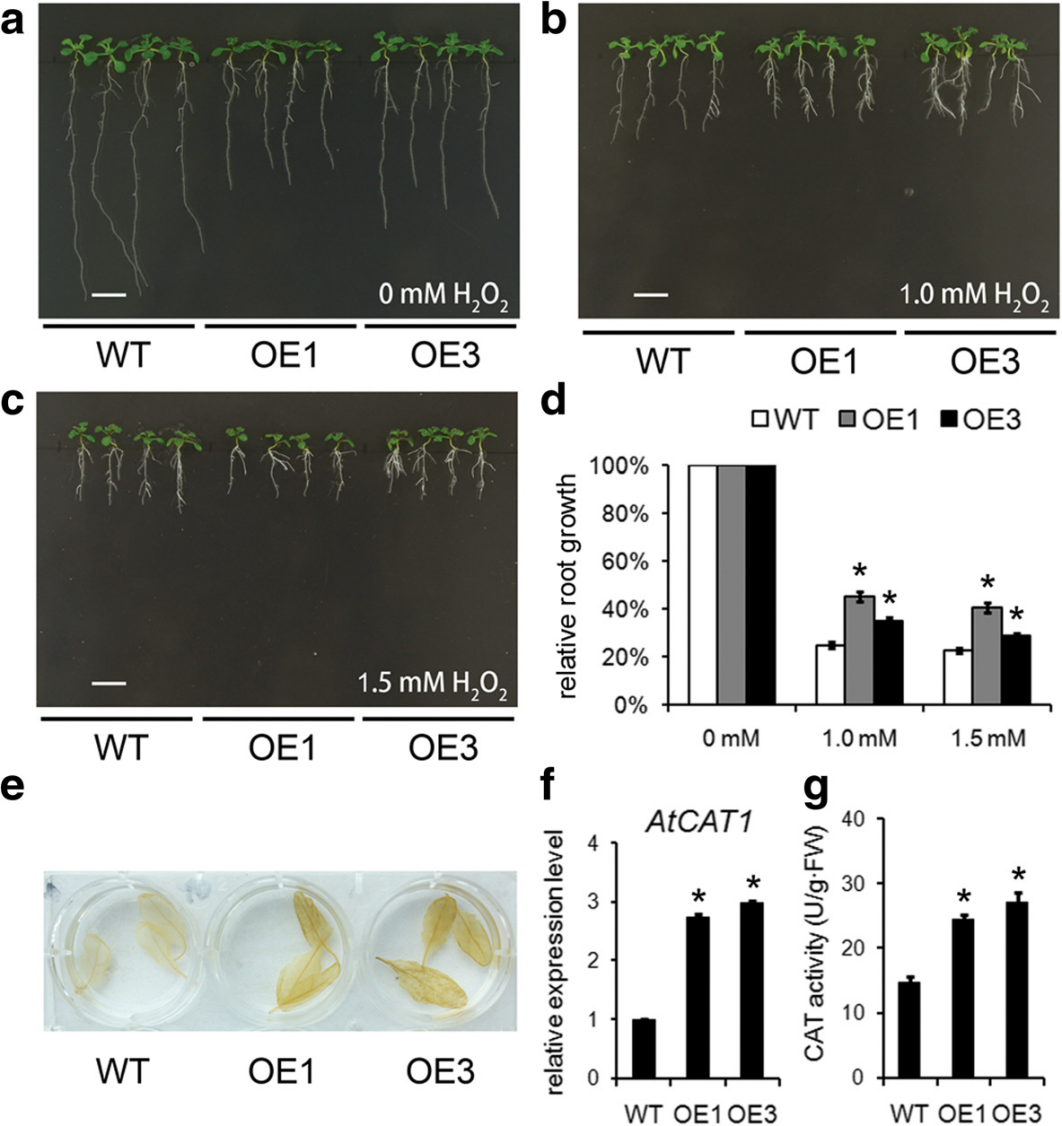
Constitutively expressing *TaBASS2* enhances ROS content and ROS scavenging activity. **a**-**c** The wild-type seedlings and two *35S::TaBASS2* transgenic lines (OE1 and OE3) after a ten-day treatment with 0, 1 or 1.5 mM H_2_O_2_. Bar = 1 cm. **d** Relative root growth of the wild-type and OE plants treated with 0, 1 or 1.5 mM H_2_O_2_. **e** DAB staining of the leaves from four-week-old soil-grown wild-type and OE plants. **f** The expression levels of *AtCAT1* in 12-day-old wild-type and OE seedlings. **g** The catalase activity in 12-day-old wild-type and OE seedlings. Error bars in (**d**) represent the standard errors (*n* = 3), with each replicate comprising 30 seedlings. Error bars in (**f**, **g**) represent the standard errors (*n* = 3), with each replicate comprising at least 12 plants. Columns labeled with an asterisk indicate means differing significantly from the WT result (*P <* 0.05, *t*-test). The expression levels were determined by RT-qPCR using *AtACT2* in *Arabidopsis* as the internal control

ROS is involved in plant response to salinity stress. To determine the role of TaBASS2 in oxidative stress response, the *35S::TaBASS2 Arabidposis* plant were assessed under H_2_O_2_ treatment. As shown in Fig. 5a, b and d, under the treatment of 1 mM H_2_O_2_, the relative root growth rate of the wild-type plants was 25 % of that under the control condition, significantly smaller than the relative root growth rates (45 % and 35 %) of OE1 and OE3, respectively. When the H_2_O_2_ concentration was raised to 1.5 mM, the relative root growth rates were 22, 40 and 29 % in the wild-type, OE1 and OE3 seedlings, respectively (Fig. 5a, c, d). These results demonstrated that the transgenic *Arabidopsis* plants constitutively expressing *TaBASS2* had higher tolerance to oxidative stress than the wild-type plants did. Moreover, treatment with methyl viologen (MV), which generates superoxide anions in plastids, demonstrated that these OE lines also displayed enhanced tolerance to plastidial oxidative stress (Additional file 6).

DAB staining results showed that both *Arabidopsis* OE lines had higher H_2_O_2_ levels in vivo than the wild-type plants (Fig. 5e). And the expression levels of the ROS-scavenging catalase 1 (*CAT1*) were also constitutively up-regulated in the transgenic *Arabidopsis* plants constitutively expressing *TaBASS2* (Fig. 5f), along with enhanced CAT1 enzyme activity (Fig. 5g). These results demonstrated that the constitutive expression of *TaBASS2* led to an increase in ROS content and ROS-scavenging activity in the transgenic *Arabidopsis* plants, suggesting constitutive activation of ROS signaling.

### The enhanced salinity tolerance in transgenic *Arabidopsis* expressing *TaBASS2* was achieved through repressing *ABI4* expression

**Fig. 6:**
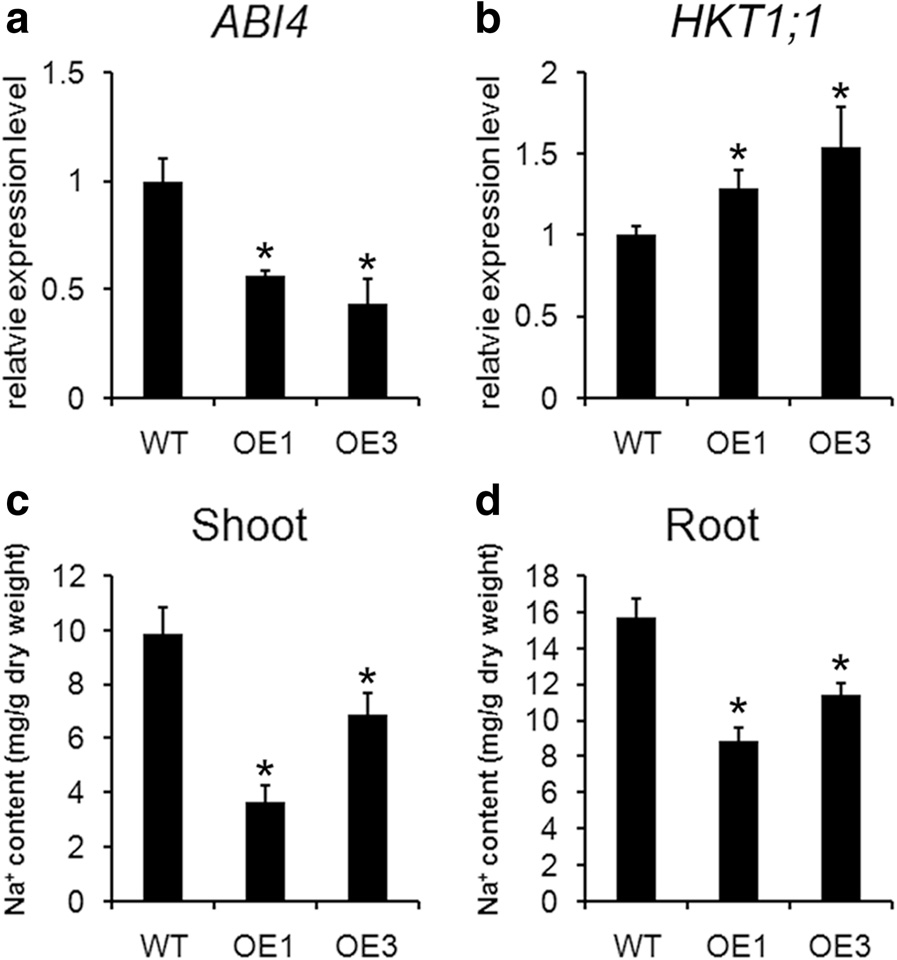
Constitutively expressing *TaBASS2* represses *ABI4* expression. **a**, **b** The expression levels of *ABI4* and *HKT1;1* in 12-day-old wild-type and two *35S::TaBASS2* transgenic lines (OE1 and OE3). Error bars represent the standard errors (*n* = 3), with each replicate comprising at least 12 plants. The expression levels were determined by RT-qPCR using *AtACT2* in *Arabidopsis* as the internal control **c**, **d** The Na^+^ contents in the shoot and root tissue from ten-day-old wild-type and OE lines. Error bars represent the standard errors (*n* = 3), with each replicate comprising at least 200 plants. Columns labeled with an asterisk indicate means differing significantly from the WT result (*P <* 0.05, *t*-test)

ABA plays an important role in response to salinity stress, so the relationship between ABA signaling and the enhanced salinity tolerance was investigated in the transgenic plants constitutively expressing *TaBASS2*. Firstly, we found the endogenous ABA contents were comparable among the wild-type *Arabidopsis* and *35S::TaBASS2* transgenic plants (Additional file 7). The expression levels of genes at the downstream of ABA-dependent stress responsive pathway were also assessed. Most of these genes, including *RESPONSIVE TO DESICCATION 29A* (*RD29A*), *RD29B*, *RD22* and *MYB2*, showed no difference in their expression levels between the wild-type and *35S::TaBASS2* plants (Additional file 8a-d). On the contrast, the expression of *ABI4*, a key component of ABA signaling pathway, was reduced by more than one fold in the *35S::TaBASS2* transgenic lines compared to the wild-type (Fig. 6a). These results demonstrated that constitutively expressing *TaBASS2* repressed *ABI4* expression without affecting ABA biosynthesis. Furthermore, the expression levels of *HKT1*, the high affinity K^+^ transporter directly regulated by ABI4 [11], were higher in two OE lines than in the wild-type plants, and the Na^+^ contents in *Arabidopsis* shoots and roots were lower in those OE lines as well (Fig. 6b-d). These results were consistent with the repression of *ABI4* expression in the transgenic plants constitutively expressing *TaBASS2*.

**Fig. 7:**
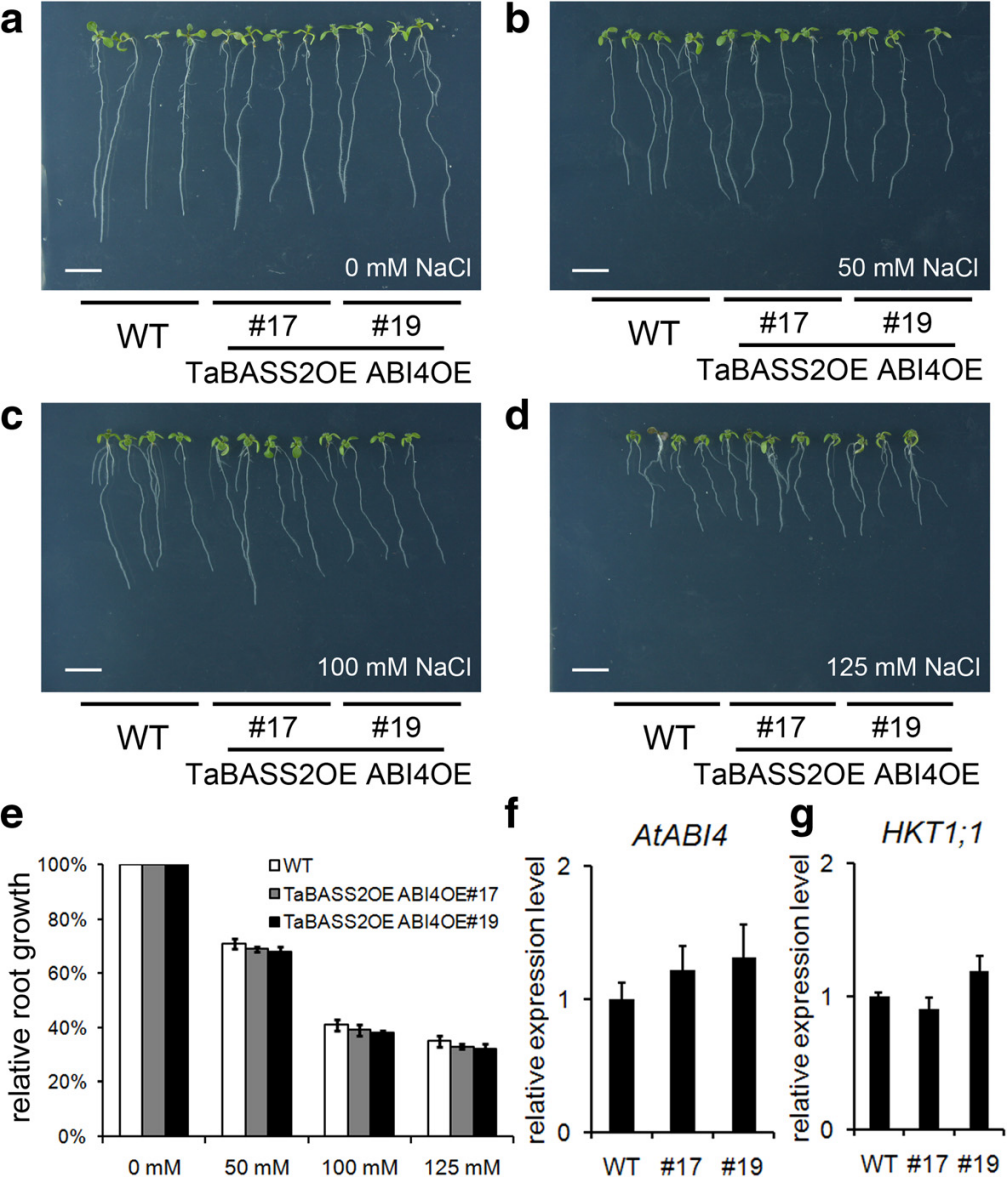
The enhanced salinity tolerance in *35S::TaBASS2 Arabidopsis* plants relies on *ABI4* suppression. **a**-**d** The wild-type seedlings and two transgenic lines constitutively expressing both *TaBASS2* and *ABI4* (TaBASS2OE ABI4OE #17 and #19) after a ten-day treatment with 0, 50, 100 or 125 mM NaCl. Bar = 1 cm. **e** Relative root growth of the wild-type and TaBASS2OE ABI4OE seedlings. Error bars represent the standard errors (*n* = 3), with each replicate comprising at least 30 seedlings. **f**, **g** The expression levels of *ABI4* (**f**) and *HKT1;1* (**g**) in the wild-type and TaBASS2OE ABI4OE seedlings. Error bars represent the standard errors (*n* = 3), with each replicate comprising at least 12 seedlings. The expression levels were determined by RT-qPCR using *AtACT2* in *Arabidopsis* as the internal control

As ABI4 was repressed in the OE plants, it was of interest to determine the salinity response when *ABI4* remained comparable expression level as in the wild-type. Thus, we carried out a genetic approach to constitutively express *ABI4* in *35S::TaBASS2* background. Two transgenic lines (TaBASS2OE ABI4OE #17 from OE1, and TaBASS2OE ABI4OE #19 from OE3) with *ABI4* expression levels comparable as in the wild type were selected for phenotypic assays (Fig. 7f). In comparison with the wild-type, both transgenic lines exhibited a similar response to NaCl treatment (Fig. 7a-e), and comparable *HKT1;1* expression levels (Fig. 7g). These results demonstrated the enhanced resistance conferred by ectopic expression of *TaBASS2* vanished when *ABI4* expression was restored, indicating that constitutively expressing *TaBASS2* in *Arabidopsis* conferred enhanced salinity tolerance by repressing *ABI4* expression.

### The transgenic wheat plants overexpressing *TaBASS2* have enhanced ROS tolerance and lower Na^+^ contents

**Fig. 8:**
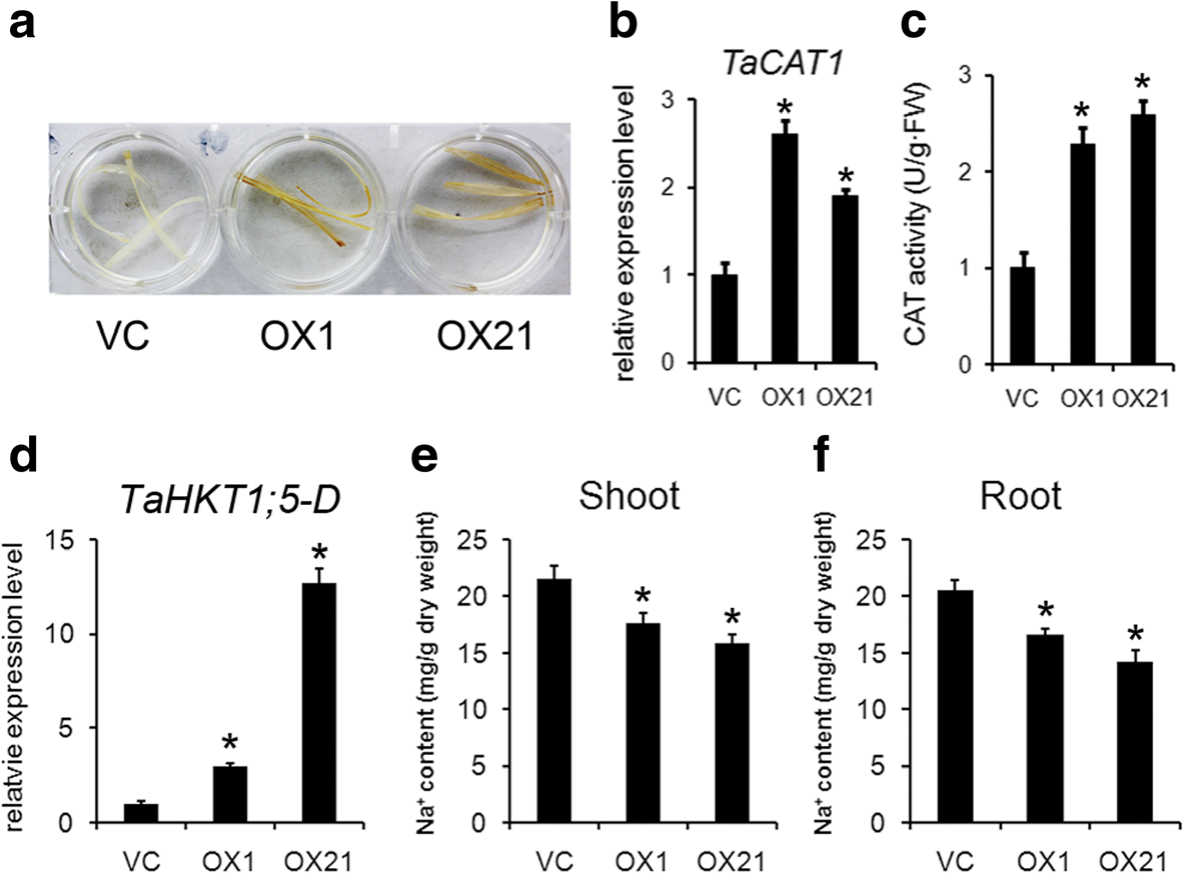
Constitutively expressing *TaBASS2* enhances ROS tolerance and reduces Na^+^ contents in wheat. **a** DAB staining of the leaves from two-week-old soil-grown vector control (VC) and two *Ubi::TaBASS2* lines (OX1 and OX21). **b** The expression levels of *TaCAT1* in two-week-old VC and OX seedlings. **c** The catalase activity in two-week-old VC and OX seedlings. **d** The expression levels of *TaHKT1;5-D* in two-week-old VC and OX seedlings. Error bars represent the standard errors (*n* = 3), with each replicate comprising at least 12 plants. The expression levels were determined by RT-qPCR using *TaCyclophil in* in wheat as the internal control. **e**, **f** The Na^+^ contents in the shoot and root tissue from two-week-old VC and OX seedlings. Error bars represent the standard errors (*n* = 3), with each replicate comprising at least 12 plants. Columns labeled with an asterisk indicate means differing significantly from the VC result (*P* < 0.05, *t*-test)

DAB staining determined wheat OX lines had higher H_2_O_2_ levels in vivo than the wild-type plants (Fig. 8a). The expression levels of the ROS-scavenging catalase 1 (*TaCAT1*) were also constitutively up-regulated in the transgenic wheat plants constitutively expressing *TaBASS2* (Fig. 8b), along with the CAT1 enzyme activity significantly higher in the transgenic lines (Fig. 8c). These results demonstrated that the constitutive expression of *TaBASS2* led to an increase in ROS contents and ROS-scavenging activity in the transgenic wheat plants, suggesting a constitutive activation of ROS signaling. The expression levels of *TaHKT1*;*5-D*, the wheat homolog of *AtHKT1*, were higher in two OX lines than in the wild-type plants (Fig. 8d). The Na^+^ contents in shoots and roots were lower in those OX lines as well (Fig. 8e and f). These results suggest that *TaHKT1;5-D* might be regulated to enhance salinity tolerance in transgenic wheat overexpressing *TaBASS2*.

## Discussion

Plastid plays a vital role in plant development, stress response, and hormone biosynthesis [24]. Since plasmid harbors its own genome, its function is orchestrated by a combination of anterograde and retrograde signaling [25–27]. The primary ABA biosynthesis pathway takes place in plastid; it starts with the methylerithrytol phosphate (MEP) pathway converting pyruvate to IPP [19]. Bile acid/sodium symporter2 (BASS2) is responsible for the transportation of pyruvate into chloroplast. Knockout of *BASS2* in *Arabidopsis* blocks pyruvate uptake into chloroplast, thus abolishes IPP synthesis in plastid, as evidenced by mevastatin-sensitive phenotype in *bass2-1* seedlings [19]. The complementation of *bass2-1* by constitutive expression of *TaBASS2* demonstrates TaBASS2 functions as a pyruvate transporter in plastid (Fig. 2). Although IPP is the precursor of ABA, its content in the plant tissue was unaffected by the constitutive expression of *TaBASS2* (Additional file 7). The marker genes in the ABA signaling pathway remained unchanged as well (Additional file 8a-d). These results established that TaBASS2 did not affect ABA biosynthesis or signaling.

ABI4 has various biological roles in plant development and stress response [6–9, 28]. Under salinity stress, ABI4 regulates ion homeostasis by its control over the expression of the sodium ion transporter gene *AtHKT1;1* [11]. In our *35S::TaBASS2* transgenic *Arabidopsis,* the expression levels of *AtHKT1;1* were constitutively up-regulated (Fig. 6), which is consistent with the previous findings of ABI4 regulating *AtHKT1;1* in salt response [11]. Moreover, the enhanced salinity tolerance was disrupted in the transgenic plants with *ABI4* expression level restored to the wild-type level (Fig. 7). Hence, the benefit of *TaBASS2* constitutive expression in salinity tolerance depends on its repression of *ABI4* expression, which suggests TaBASS2 participates in salt response through regulating *ABI4* expression. Because *ABI4* homolog has not been identified in wheat, it cannot be tested if TaBASS2 regulates such a signaling node in wheat response to salinity stress. However, we found up-regulation of *TaHKT1;5-D*, the homolog of *AtHKT1;1*, as well as reduced Na^+^ concentration in the transgenic wheat seedlings overexpressing *TaBASS2* (Fig. 8). These results together suggest a similar mechanism as in *Arabidopsis*. Besides regulation of *ABI4* expression, the constitutive expression of *TaBASS2* resulted in increased tissue ROS contents in both wheat and *A. thaliana*, as did the activity of catalase and the transcription level of its encoding gene *CAT1* (Figs. 5 and 8). As a result, the transgenic lines exhibited not just superior salinity tolerance, but also improved tolerance to oxidative stress (Fig. 5, Additional file 6).

How TaBASS2, a plastidial pyruvate symporter, functions in salinity tolerance remains an interesting question. The elevated ROS contents in *TaBASS2* overexpression plants suggest the involvement of ROS signaling in TaBASS2 function (Fig. 5). Recent studies have shown that ROS not only causes oxidative stress in plant cells, but serves as potential signals in the PET retrograde signaling pathway [16]. ROS accumulation triggers a series of stress-responsive genes, inducing ROS scavenging activity and thereby contributing to the plant’s redox homeostasis [8]. Coupled function of RbohD-derived ROS production and plastid hemeoxygenases in salinity response strongly suggests that the chloroplast-to-nucleus retrograde signaling is involved in plant salinity response [29, 30]. In retrograde signaling pathway, ABI4 serves as a node in the tetrapyrrole and plastid gene expression (PGE). The PGE marker genes *LIGHT-HARVESTING CHLOROPHYLL A/B-BINDING PROTEIN* (*LHCB*) and *RUBISCO SMALL SUBUNIT* (*RBCS*) are suppressed by retrograde pathway’s activator, norflurazone and lincomycin, while in *Arabidposis abi4* mutant these marker genes expression were de-repressed [8]. Here, we also found these two genes were less suppressed in *35S::TaBASS2* plants than the wild-type (Additional file 9), indicating that both pathways were affected by the constitutive expression of TaBASS2. The transcription levels of genes in MEcPP pathway, such as *MEcPP SYNTHASE* (*MDS*) and *HYDROPEROXIDE LYASE* (*HPL*), were comparable between the *35S::TaBASS2* and the wild-type plants (Additional file 8e and f). Taken together, these results suggest that ectopic expression of *TaBASS2* in Arabidopsis regulates retrograde signaling by repressing *ABI4* expression.

## Conclusions

Here in this study, we characterized a putative plastidal pyruvate transporter, TaBASS2, in wheat salinity response. Constitutive expression of *TaBASS2* enhanced salinity tolerance in both transgenic wheat and *Arabidopsis*, accompanied with elevated ROS contents and repression in *ABI4* expression. As ROS and ABI4 play crucial roles in plastid-nucleus retrograde signaling, our findings also suggest that TaBASS2 modulates retrograde signaling to positively regulate plant response to salinity stress.

## Methods

### Wheat growing conditions and stress treatments

Two cultivars of bread wheat (*Triticum aestivum*), Shanrong3 (SR3) and Yangmai20 (YM20), were used in this study. We previously bred SR3 with high salt tolerance from a wheat introgression hybrid, which was constructed with a common wheat cultivar Jinan 177 (with modest salt tolerance) as the recipient and tall wheatgrass (*Thinopyrum elongatum*, wheat’s close relative, one of the monocots with highest salt tolerance) as the donor via asymmetric somatic hybridization [21]. YM20, with modest salt tolerance, was bred by Jiangsu Lixiahe Institute of Agricultural Science. Wheat plants were grown in a controlled greenhouse on campus. Wheat plants were grown in half strength Hoagland’s liquid medium at 22 °C under a 16-h-light/8-h-dark photoperiod. For stress treatments, the three-leaf-stage seedlings were treated with 200 mM NaCl, 10 mM H_2_O_2_ or 100 μM ABA. The root tissue was harvest after a 48-h treatment for RNA extraction. The phenotypic effects of salinity stress on 10-day-old seedlings were scored after a four-day NaCl treatment with concentration increasing by 50 mM and another four-day treatment with 200 mM NaCl treatment, as previously described [31].

*Arabidopsis thaliana* Columbia-0 (Col-0) is used in this study as the wild-type. The *bass2-1* mutant (SALK_101808C) was purchased from Arabidopsis Biological Resource Center. *Arabidopsis* seeds were surface-sterilized with 70 % (v/v) ethanol, then placed on Murashige and Skoog (MS) agar plates, which were incubated in the dark at 4 °C for three days before moved to a 22 °C growth chamber with a relative humidity of 70 % and a 16-h light/8-h dark photoperiod (light intensity 200 μM · m^-2^ · s^-1^). For stress treatments, four-day-old seedlings were transferred onto a MS plate containing 0, 50, 100, 125 mM NaCl; 0, 1, 1.5 mM H_2_O_2_; or 0, 1, 1.5 mM methyl viologen (MV) for ten days. Primary root lengths were measured from digitized images in the Image J software (http://imagej.nih.gov/ij/). Another salinity stress treatment was conducted on the four-week-old soil-grown *Arabidopsis* plants by adding 50 mM NaCl for three days, 100 mM for three days, 150 mM for three days and 200 mM for five days, as previously described [32]. The plants were allowed to grow for another two weeks before their survival rates were calculated. For mevastatin treatment, four-day-old seedlings were transferred to the plates supplemented with 0 nM or 500 nM mevastatin and grown vertically for another seven days.

### *TaBASS2* isolation, sequence characterization and transformation

The fragment of the *TaBASS2* sequence identified in the previous microarray was used to search wheat expression sequence tags (http://www.ncbi.nlm.nih.gov/nucest/?term=wheat) [23]. The resulting hits were assembled using CAP3 software [33], and the entire *TaBASS2* coding region was cloned using a pair of primers (5′-ATG GCG CCT TCC GCG ACC TGC C-3′/5′-TCA TTC CTT GAA ATC GTC CTT G-3′) designed from the reconstructed sequence. The predicted protein sequence was aligned with other BASS2 sequences using the CLUSTALW2 algorithm (www.ebi.ac.uk/Tools/msa/clustalw2/), and a phylogenetic analysis was conducted using the neighbor-joining method implemented in the MEGA4 software [34]. To generate transgenic wheat plants, the *TaBASS2* coding sequence was ligated into pGA3626 driven by a maize ubiquitin promoter and introduced into wheat cv. YM20 using the shoot apical meristem method [35]. To generate transgenic *Arabidopsis*, the *TaBASS2* coding sequence was ligated into pROK2, and *ABI4* into pJIM19 (transgene driven by a 35S promoter). The cassette containing *TaBASS2* was introduced into either *A. thaliana* ecotype Col-0 or the knockout mutant *bass2-1* (SALK_101808C), and the cassette containing the *ABI4* coding sequence was introduced into *TaBASS2* constitutive expressors using the floral dip method [36]. Transformants were selected as previously [32], and homozygous T_3_ progeny was used in the subsequent experiments.

### Subcellular localization of TaBASS2

The *TaBASS2* coding sequence was inserted into a pBI221 vector with eGFP sequence in frame. TaBASS2-GFP fusion protein was transiently expressed in *A. thaliana* mesophyll protoplasts using a PEG-mediated transformation method [37]. The protoplasts were then incubated in the dark for 16 h at 25 °C before observed under a confocal laser scanning microscopy (Zeiss, Oberkochen, Germany).

### Reverse Transcriptase Quantitative PCR

Total RNA was extracted from plant tissues with the TRIzol reagent (Invitrogen, Carlsbad, CA, USA), followed by cDNA synthesis from 2 μg RNA with the SuperScript II reverse transcriptase (Invitrogen). Three biological replicates were included for each assay. Reverse Transcriptase Quantitative PCR (RT-qPCR) was conducted with the FastStart Universal SYBR Green Master (Roche, Basel, Switzerland) on an Eppendorf Mastercycler RT-qPCR device (Eppendorf, Hamburg, Germany). The gene relative expression levels were calculated using the 2^-ΔΔCT^ method [38]. A cyclophilin (AF384147) gene and *Actin2* (*At3g18780*) were used as the internal controls in wheat and *Arabidopsis*, respectively. The RT-qPCR primers are listed in Additional file 10.

### Measurement of Na^+^ concentration

The root and shoot tissues were harvest from the ten-day-old *Arabidopsis* seedlings grown on MS plates, washed with distilled water for 5 times, dried at 65 °C for 4 days and digested in 6 M hydrochloric acid solution before the assay, as previously described [39]. The Na^+^ concentrations were determined by a Thermo Iris Intrepid II Inductively Coupled Plasma Atomic Emission Spectrometer (ICP; Thermo Electron Corporation, Franklin, MA).

### Determination of peroxide level, catalase activity and ABA content

H_2_O_2_ levels in the leaves were determined by the DAB staining of four-week-old soil-grown *Arabidopsis* plants [40]. Catalase activities were measured in ten-day-old *Arabidopsis* seedlings with a commercial kit purchased from Beyotime Institute of Biotechnology (Haimen, China). ABA contents in ten-day-old seedlings were assessed as described previously by LC-MS-MS [31].

### Ethics

Not applicable.

### Consent to publish

Not applicable.

### Availability of data and materials

The phylogenetic data shown in Additional file 1b was deposited into a public phylogenetic database, Treebase.org, with the link (http://purl.org/phylo/treebase/phylows/study/TB2:S19163). The datasets supporting the conclusions of this article are included within the article and its additional files.

## Additional files

Additional file 1: Figure S1. Multi-alignment and phylogenetic analysis of BASS proteins. (a) Multi-alignment of six BASS2 protein sequences. The transmembrane domains (TM1-8) are indicated. The barley HvBASS2 (BAJ88629.1), rice OsBASS2 (NP_917201.1), sorghum SbBASS2 (NP_917201.1), *Flaveria trinervia* FtBASS2 (BAJ16226.1), and *Arabidopsis* BASS2 (NP_850089) were used for the multi-alignment. (b) Phylogenetic analysis of 42 BASS proteins. For each sequence, either the gene ID or GI number was shown. (TIF 236 kb) Additional file 2: Figure S2. Subcellular localization of TaBASS2 in *Arabidopsis* protoplasts. Bar = 10 μm. (TIF 346 kb) Additional file 3: Figure S3. Shoot (a) and root (b) length of 20-day-old wheat vector control (VC) and *TaBASS2* overexpression (OX) seedlings. Error bars represent the standard errors (*n =* 3), with each replicate comprising at least 30 plants. Columns labeled with an asterisk indicate means differing significantly from the VC result (*P <* 0.05, *t*-test). (TIF 67 kb) Additional file 4: Figure S4. The root lengths of the wild-type and two *35S::TaBASS2* transgenic lines (OE1 and OE3) after treatment with 0, 50, 100, 150 mM NaCl (a), 0, 1, 1.5 mM H_2_O_2_ (b), or 0, 1, 1.5 mM methyl viologen (MV) (c). (TIF 52 kb) Additional file 5: Figure S5. Salinity tolerance in *Arabidopsis bass2-1* mutant is comparable with the wild-type plants*.* (a) Survival rates of four-week-old soil-grown wild-type and *bass2-1* plants measured 14 days after NaCl treatment. Error bars represent the standard errors (*n* = 3), with each replicate comprising at least 30 plants. (b, c) The expression levels of *ABI4* (b) and *HKT1;1* (c) in 12-day-old wild-type and *bass2-1*. (TIF 55 kb) Additional file 6: Figure S6. Constitutively expressing *TaBASS2* enhances tolerance to methyl viologen (MV). (a-c) The wild-type seedlings and two *35S::TaBASS2* transgenic lines (OE1 and OE3) after a ten-day treatment with 0, 1 or 1.5 mM MV. Bar = 1 cm. (d) Relative root growth of the wild-type and OE plants treated with 0, 1 or 1.5 mM MV. Error bars represent the standard errors (*n* = 3), with each replicate comprising at least 30 plants. Columns labeled with an asterisk indicate means differing significantly from the WT result (*P <* 0.05, *t*-test). (TIF 2419 kb) Additional file 7: Figure S7. The ABA contents of ten-day-old wild-type, two *35S::TaBASS2* transgenic lines (OE1 and OE3), and *bass2-1Arabidopsis* seedlings. Error bars represent the standard errors (*n =* 3), with each replicate comprising at least 12 seedlings. (TIF 69 kb) Additional file 8: Figure S8. The expression levels of ABA and MEcPP markers in ten-day-old seedlings of WT and and two *35S::TaBASS2* transgenic lines (OE1 and OE3). Error bars represent the standard errors (*n* = 3), with each replicate comprising at least 12 seedlings. The expression levels were determined by RT-qPCR using *AtACT2* in *Arabidopsis* as the internal control. (TIF 54 kb) Additional file 9: Figure S9. The expression levels of PGE retrograde signaling branch markers, *AtRBCS*, *AtLHCB1.1*, and *AtLHCB2.4* in three-day-old wild-type and two *35S::TaBASS2* transgenic lines (OE1 and OE3) treated with lincomycin (a) or norflurazone (b). (TIF 72 kb) Additional file 10: Table S1. PCR primer sequences used in this study. (DOCX 17 kb)
