# Supplementary figures and images for: A putative pyruvate transporter TaBASS2 positively regulates salinity tolerance in wheat via modulation of ABI4 expression
Source: BMC Plant Biol. 2016 May 10;16:109. doi: 10.1186/s12870-016-0795-3 (PMC4862123; doi:10.1186/s12870-016-0795-3)

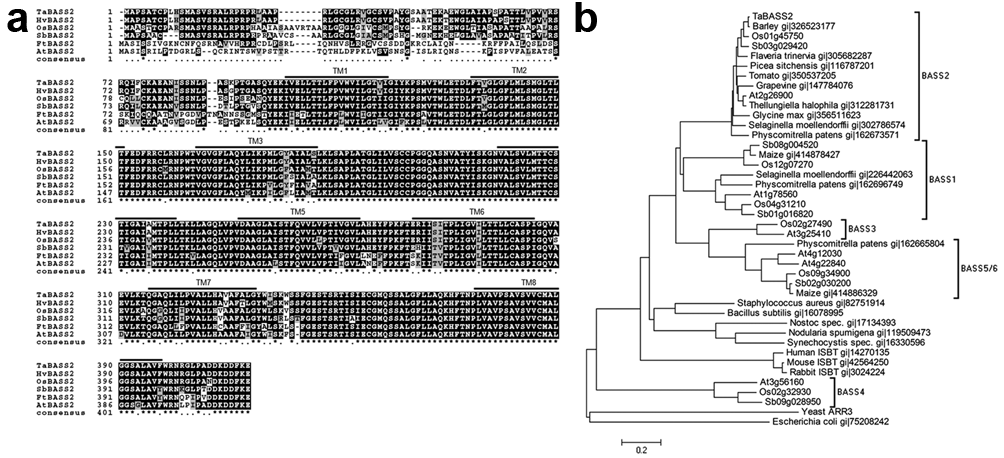

Supplement: Additional file 1: Figure S1. — Multi-alignment and phylogenetic analysis of BASS proteins. (a) Multi-alignment of six BASS2 protein sequences. The transmembrane domains (TM1-8) are indicated. The barley HvBASS2 (BAJ88629.1), rice OsBASS2 (NP_917201.1), sorghum SbBASS2 (NP_917201.1), Flaveria trinervia FtBASS2 (BAJ16226.1), and Arabidopsis BASS2 (NP_850089) were used for the multi-alignment. (b) Phylogenetic analysis of 42 BASS proteins. For each sequence, either the gene ID or GI number was shown. (TIF 236 kb) [file 12870_2016_795_MOESM1_ESM.tif]

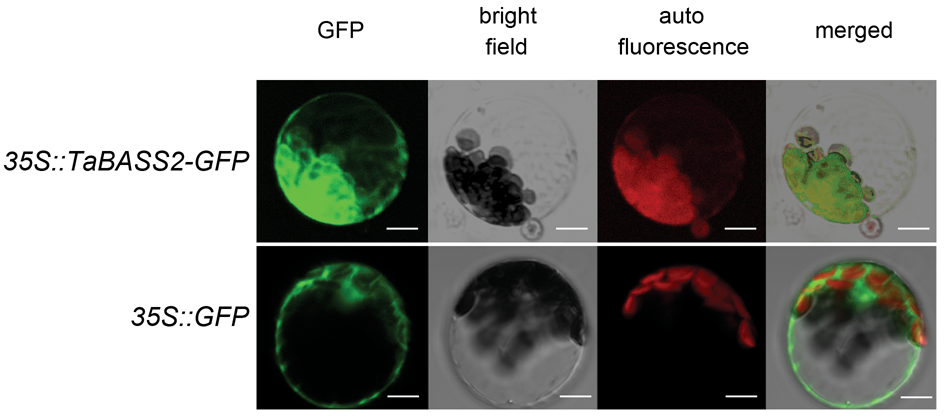

Supplement: Additional file 2: Figure S2. — Subcellular localization of TaBASS2 in Arabidopsis protoplasts. Bar = 10 μm. (TIF 346 kb) [file 12870_2016_795_MOESM2_ESM.tif]

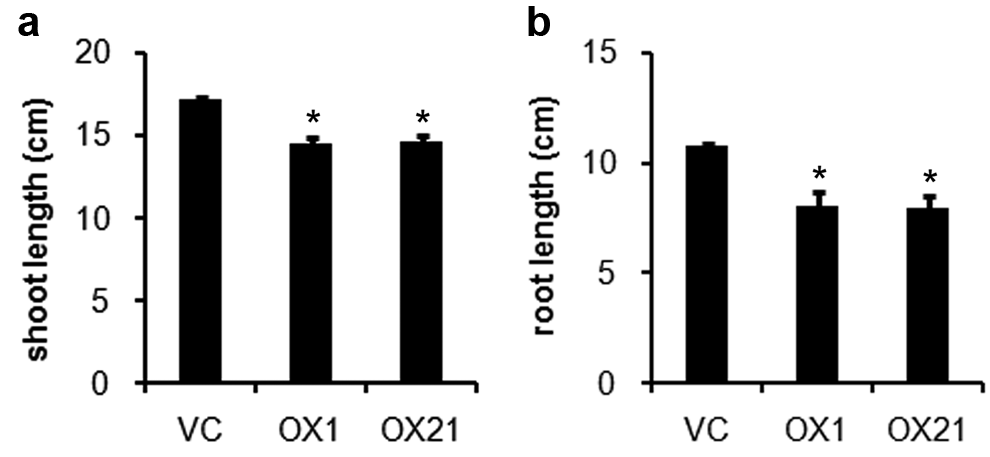

Supplement: Additional file 3: Figure S3. — Shoot (a) and root (b) length of 20-day-old wheat vector control (VC) and TaBASS2 overexpression (OX) seedlings. Error bars represent the standard errors (n = 3), with each replicate comprising at least 30 plants. Columns labeled with an asterisk indicate means differing significantly from the VC result (P < 0.05, t-test). (TIF 67 kb) [file 12870_2016_795_MOESM3_ESM.tif]

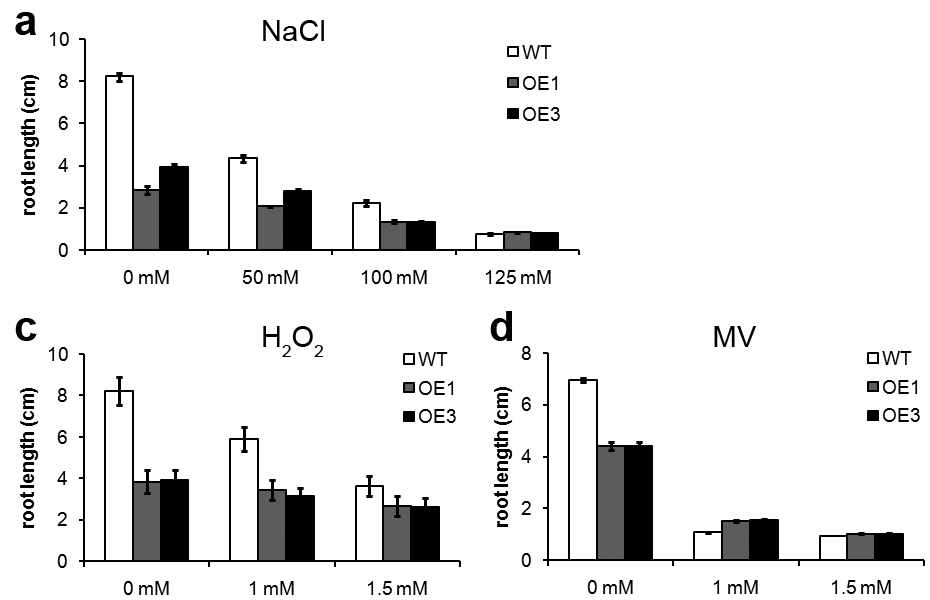

Supplement: Additional file 4: Figure S4. — The root lengths of the wild-type and two 35S::TaBASS2 transgenic lines (OE1 and OE3) after treatment with 0, 50, 100, 150 mM NaCl (a), 0, 1, 1.5 mM H2O2 (b), or 0, 1, 1.5 mM methyl viologen (MV) (c). (TIF 52 kb) [file 12870_2016_795_MOESM4_ESM.tif]

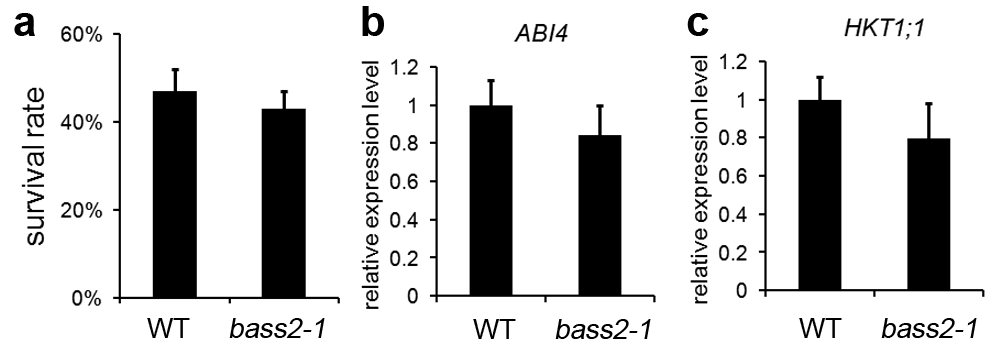

Supplement: Additional file 5: Figure S5. — Salinity tolerance in Arabidopsis bass2-1 mutant is comparable with the wild-type plants. (a) Survival rates of four-week-old soil-grown wild-type and bass2-1 plants measured 14 days after NaCl treatment. Error bars represent the standard errors (n = 3), with each replicate comprising at least 30 plants. (b, c) The expression levels of ABI4 (b) and HKT1;1 (c) in 12-day-old wild-type and bass2-1. (TIF 55 kb) [file 12870_2016_795_MOESM5_ESM.tif]

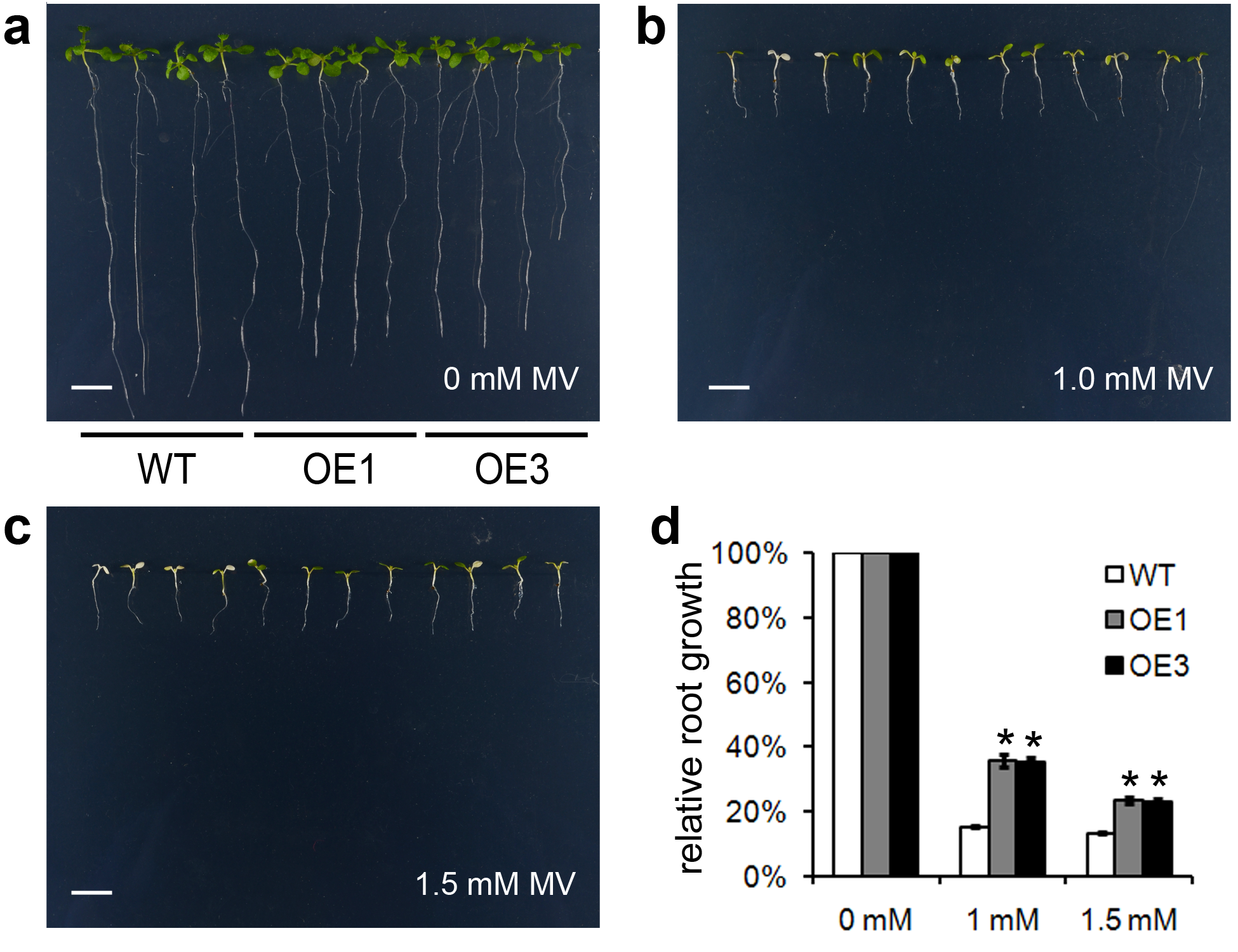

Supplement: Additional file 6: Figure S6. — Constitutively expressing TaBASS2 enhances tolerance to methyl viologen (MV). (a-c) The wild-type seedlings and two 35S::TaBASS2 transgenic lines (OE1 and OE3) after a ten-day treatment with 0, 1 or 1.5 mM MV. Bar = 1 cm. (d) Relative root growth of the wild-type and OE plants treated with 0, 1 or 1.5 mM MV. Error bars represent the standard errors (n = 3), with each replicate comprising at least 30 plants. Columns labeled with an asterisk indicate means differing significantly from the WT result (P < 0.05, t-test). (TIF 2419 kb) [file 12870_2016_795_MOESM6_ESM.tif]

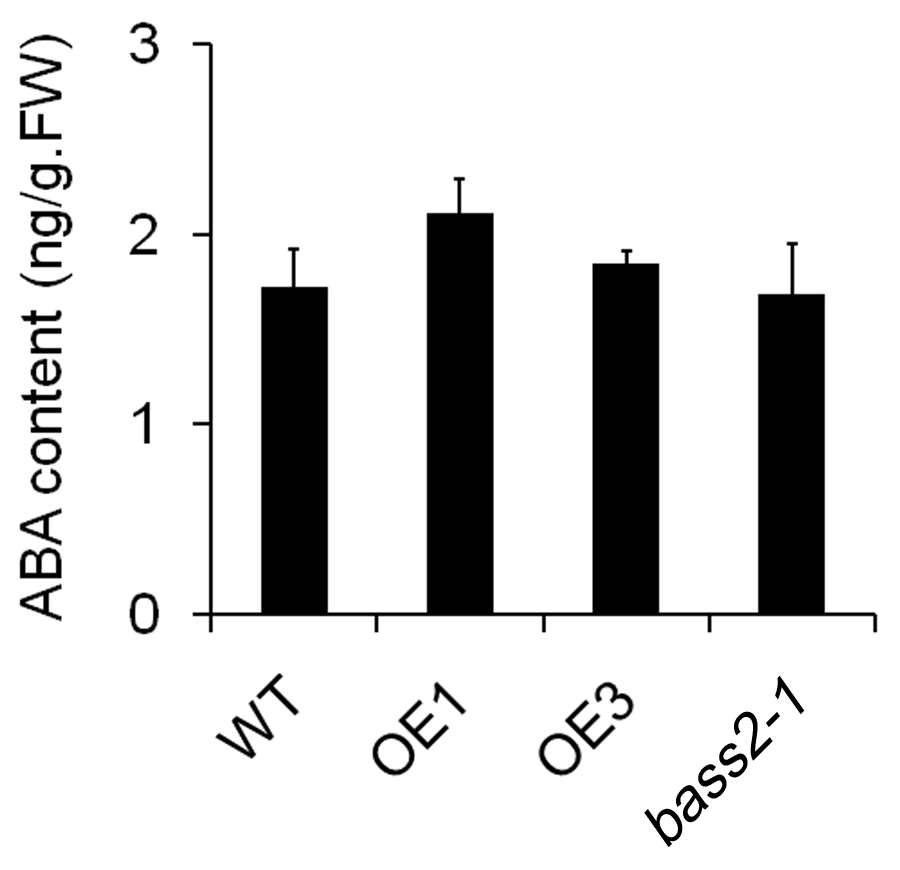

Supplement: Additional file 7: Figure S7. — The ABA contents of ten-day-old wild-type, two 35S::TaBASS2 transgenic lines (OE1 and OE3), and bass2-1Arabidopsis seedlings. Error bars represent the standard errors (n = 3), with each replicate comprising at least 12 seedlings. (TIF 69 kb) [file 12870_2016_795_MOESM7_ESM.tif]

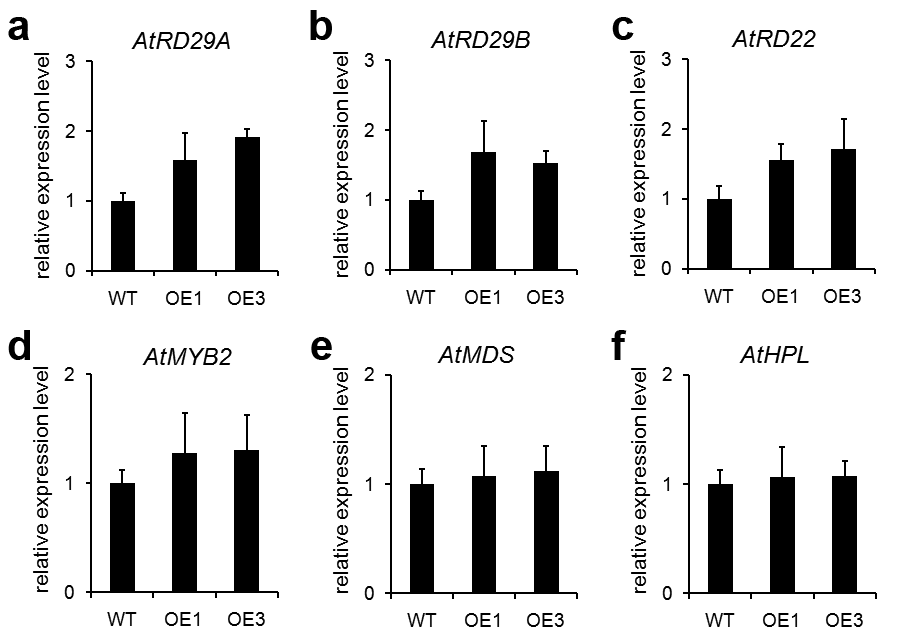

Supplement: Additional file 8: Figure S8. — The expression levels of ABA and MEcPP markers in ten-day-old seedlings of WT and and two 35S::TaBASS2 transgenic lines (OE1 and OE3). Error bars represent the standard errors (n = 3), with each replicate comprising at least 12 seedlings. The expression levels were determined by RT-qPCR using AtACT2 in Arabidopsis as the internal control. (TIF 54 kb) [file 12870_2016_795_MOESM8_ESM.tif]

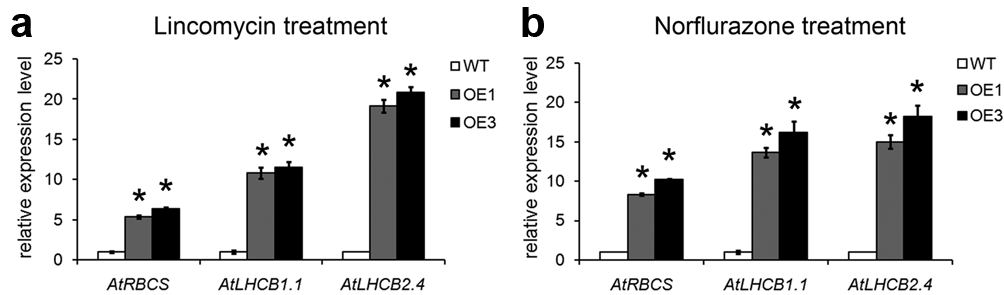

Supplement: Additional file 9: Figure S9. — The expression levels of PGE retrograde signaling branch markers, AtRBCS, AtLHCB1.1, and AtLHCB2.4 in three-day-old wild-type and two 35S::TaBASS2 transgenic lines (OE1 and OE3) treated with lincomycin (a) or norflurazone (b). (TIF 72 kb) [file 12870_2016_795_MOESM9_ESM.tif]
