# Supplementary material for: A putative pyruvate transporter TaBASS2 positively regulates salinity tolerance in wheat via modulation of ABI4 expression
Source: BMC Plant Biol. 2016 May 10;16:109. doi: 10.1186/s12870-016-0795-3 (PMC4862123; doi:10.1186/s12870-016-0795-3)
Supplement: Additional file 10: Table S1. — PCR primer sequences used in this study. (DOCX 17 kb) [file 12870_2016_795_MOESM10_ESM.docx]

**Table S1.**PCR primer sequences used in this study.

| Name | Accession | Forward primer | | Reverse primer |
| --- | --- | --- | --- | --- |
| *TaCyclophilin* | *AF384147* | ACGGCTCCCAGTTCTTCA | GCCCACCTTCTCGATGTTC | |
| *TaBASS2* | *KP638564* | ATAGCGATGACACCACTCCT | TTTCAACACTTCTGCGACTT | |
| *TaCAT* | *GU984379* | TGCTGCAGACAAGGATCTTC | TCGTAGTGGTTGTTGTGGTG | |
| *TaHKT1;5-D* | *DQ646342* | CTGCGGCTTCGTCCCGA | CGCTAGCACGAACGCCG | |
| *AtACT2* | *At3g18780* | TTGACTACGAGCAGGAGATGG | ACAAACGAGGGCTGGAACAAG | |
| *AtBASS2* | *At2g26900* | TTCCGGCAGATGACAAGGATGACT | TGTCATCATGCACGCCCTTGTTTC | |
| *AtABI4* | *At2g40220* | TTACCGTGGCGTTCGACAA | GAGTGCGCTTACGTGGCTCT | |
| *AtCAT1* | *At1g20630* | CGCCGATTTGCGAGATACACACAG | GACCTCGAGTTCCGACAGTCAAAGA | |
| *HKT1;1* | *At4g10310* | TCAGTGCATATGGAAACGTTGG | CAGCCACCATCGCTGATGm | |
| *MDS* | *At1g63970* | CGAAGAAGAACGGCAATGGCTACT | CGAGGCTGAAGGACGACATGATAAC | |
| *HPL* | *At4g15440* | GCTGAGAACGGTTGGAAAAC | TCCGGCGATTAAGAGAGAAG | |
| *RBCS* | *At1g67090* | GTGTTGGGTTCAAAGCTGGT | CTCGGAATGCTGCCAAGATA | |
| *LHCB1.1* | *At1g29920* | GATTGGCTATGTTCTCTATGTTTTG | AAATGGTCAGCAAGGTTCTCTATC | |
| *LHCB2.4* | *At3g27690* | GCCATCCAACGATCTCCTC | TGGTCCGTACCAGATGCTC | |
| *RD29A* | *At5g52310* | CTTGATGGTCAACGGAAGGT | CAATCTCCGGTACTCCTCCA | |
| *RD29B* | *At5g52300* | AGAAGGAATGGTGGGGAAAG | CAACTCACTTCCACCGGAAT | |
| *RD22* | *At5g25610* | AGGAGCAAACCCTTTCGTGT | CGTTTCAACGTCTCCGAAAA | |
| *MYB2* | *At2g47190* | AACGTCTTCGAATTCTCCGGCTGA | ATCGTTGAACTCTCCGAAACCCGT | |
